# Spatiotemporal variation of potential evapotranspiration and its dominant factors during 1970−2020 across the Sichuan-Chongqing region, China

**DOI:** 10.1371/journal.pone.0268702

**Published:** 2022-06-24

**Authors:** Qingzhou Zheng, Jun He, Mengsheng Qin, Xia Wu, Tiantian Liu, Xiaolin Huang

**Affiliations:** 1 Chongqing Meteorology Bureau, Chongqing, China; 2 Yangzhou Meteorology Bureau, Yangzhou, China; 3 Baotou Meteorology Bureau, Baotou, China; 4 Nanjing University of Information Science & Technology, Nanjing, China; Universiti Teknologi Malaysia, MALAYSIA

## Abstract

Analyzing the primary factors of potential evapotranspiration (PET) dynamic is fundamental to accurately estimating crop yield, evaluating environmental impacts, and understanding water and carbon cycles. Previous studies have focused on regionally average regional PET and its dominant factors. Spatial distributions of PET trends and their main causes have not been fully investigated. The Mann–Kendall test was used to determine the significance of long-term trends in PET and five meteorological factors (net radiation, wind speed, air temperature, vapor pressure deficit, relative humidity) at 56 meteorological stations in the Sichuan-Chongqing region from 1970 to 2020. Furthermore, this present study combining and quantitatively illustrated sensitivities and contributions of the meteorological factors to change in annual and seasonal PET. There was a positive trend in PET for approximately 58%, 68%, 38%, 73% and 73% of all surveyed stations at annual, spring, summer, autumn and winter, respectively. Contribution analysis exhibited that the driving factors for the PET variation varied spatially and seasonally. For stations with an upward PET trend, vapor pressure deficit was a dominant factor at all time scales. For stations with a downward PET trend, annual changes in PET mainly resulted from decreased wind speed, as did changes in spring, autumn and winter; decreasing net radiation was the dominant factor in summer. The positive effect of the vapor pressure deficit offset the negative effects of wind speed and net radiation, leading to the increasing PET in this area as a whole. Sensitivity analysis showed that net radiation and relative humidity were the two most sensitive variables for PET, followed by vapor pressure deficit in this study area. Results from the two mathematical approaches were not perfect match, because the change magnitude of the meteorological factors is also responsible for the effects of meteorological factors on PET variation to some extent. However, conducting sensitivity and contribution analysis in this study can avoid the uncertainties from using a single method and provides detailed and well-understood information for interpreting the influence of global climate change on the water cycle and improving local water management.

## 1 Introduction

It is widely accepted that climate change and human activities profoundly affect local water energy transfer and water balance processes, such as evapotranspiration (ET) [1, 2]. Changes in these processes can have serious consequences, including increased risk of flooding, exacerbated urban heat island and dry island effects, and deterioration of water quality [3, 4]. ET, together with precipitation and runoff, is a fundamental component of the hydrological cycle [5–7]. ET is important in energy, water and carbon cycles and is a key climate parameter when analyzing regional-scale activity of hydroclimatic change and water cycle [8, 9].

Potential evapotranspiration (PET) is water lost to the atmosphere from well-watered crops under optimum soil water conditions [3, 10]. The meteorological variables are the only factors impacting PET so that PET can be calculated from weather data [10, 11]. The Penman-Monteith (P-M) equation is a standard model for estimating PET that is recommended by the United Nations Food and Agriculture Organization (FAO) [10]. The P-M equation includes climate variables such as air temperature (*T*_*a*_), net radiation (*R*_*n*_) and wind speed (*W*_*s*_) and is appropriate for humid conditions [7, 12–14]. The effects of different source parameters on PET have been investigated over different periods and in different regions. Many studies indicated that the decreased W_s_ was mainly responsible for the decline of PET [7, 14, 15]. Another widely accepted explanation for the PET reduction is the decrease in solar radiation caused by increasing cloud cover [13, 16]. Moreover, many researchers have identified drivers of increases in PET. Some researchers revealed that the relative humidity (RH) was the decisive factor of the increase in annual PET in many regions, such as Wei River basin [6] and Zhejiang province [17]. Meanwhile, some other researchers attributed the elevated PET to the increased vapor pressure deficit (VPD) [4, 11, 18, 19]. Climate change is highly heterogeneous owing to differences in the study period and study regions. Therefore, future studies are needed to evaluate regional-scale change in PET and its dominant factors explicitly.

This study was carried out in the Sichuan–Chongqing (SC-CQ) region, located in the east of the Qinghai–Tibet Plateau [20–22]. This region was characterized by diverse geological and climatic features, resulting in uneven distributions of climate elements and natural resources [23, 24]. The SC-CQ region contains two megacities (Chengdu and Chongqing), and has a total population of 115.7 million [22, 23]. The Chinese government launched the national strategy for the development of western regions in 2000. Therefore, the SC-CQ region became the core growth area of Southwest China and has experienced dramatic urban expansion and population growth in recent decades, which has directly affected local weather and hydrology [25]. In areas with rapid urbanization and industrialization, the hydrologic cycle is sensitive to human activities and is more prone to climate change than other areas [3, 5]. Recently, serious water-related issues have led to increasing problems in the eco-hydrological environment in this region [21]. Disastrous intense floods, extreme droughts and a series of severe environmental and ecological problems have affected this area in recent times, which attracts wide attention to disaster mitigation and prevention [20, 26, 27]. In this context, identification of trends in PET and its contributing factors will enhance our understanding of the effects of climate change on future water balance and thus enable the formulation of effective policies for water resource management in this region [4, 5, 15].

Spatial heterogeneity in PET and its causal mechanisms are to be expected in the study area due to the changeable climate and the complex topography. Previous literature has only focused on average regional PET and climatic elements of a long record. The spatial distributions of drivers of PET have not been adequately considered, which may cause deviation of regional PET from the real situation [15]. Understanding the spatial differences of PET is of great importance for identifying the mechanisms and processes by which environmental dynamics and anthropogenic activities influence regional hydrology in the study area [3, 5, 15]. Thus, more attention should be paid to the PET research on spatial patterns of the stations instead of regional averaging.

The aims of this study were: (1) to identify temporal and spatial variation in PET across the SC-CQ region from 1970 to 2020; (2) to identify spatial and seasonal differences in the driving factors of PET; and (3) to quantify the contributions and sensitivity of dominant climate factors to PET changes. This study presents a detailed investigation of the heterogeneous spatial distribution of PET dynamics and the associated contributing meteorological factors using advanced statistical methods and geographic information systems (GIS).

## 2 Data and methods

### 2.1 Study area and data sources

**Fig 1:**
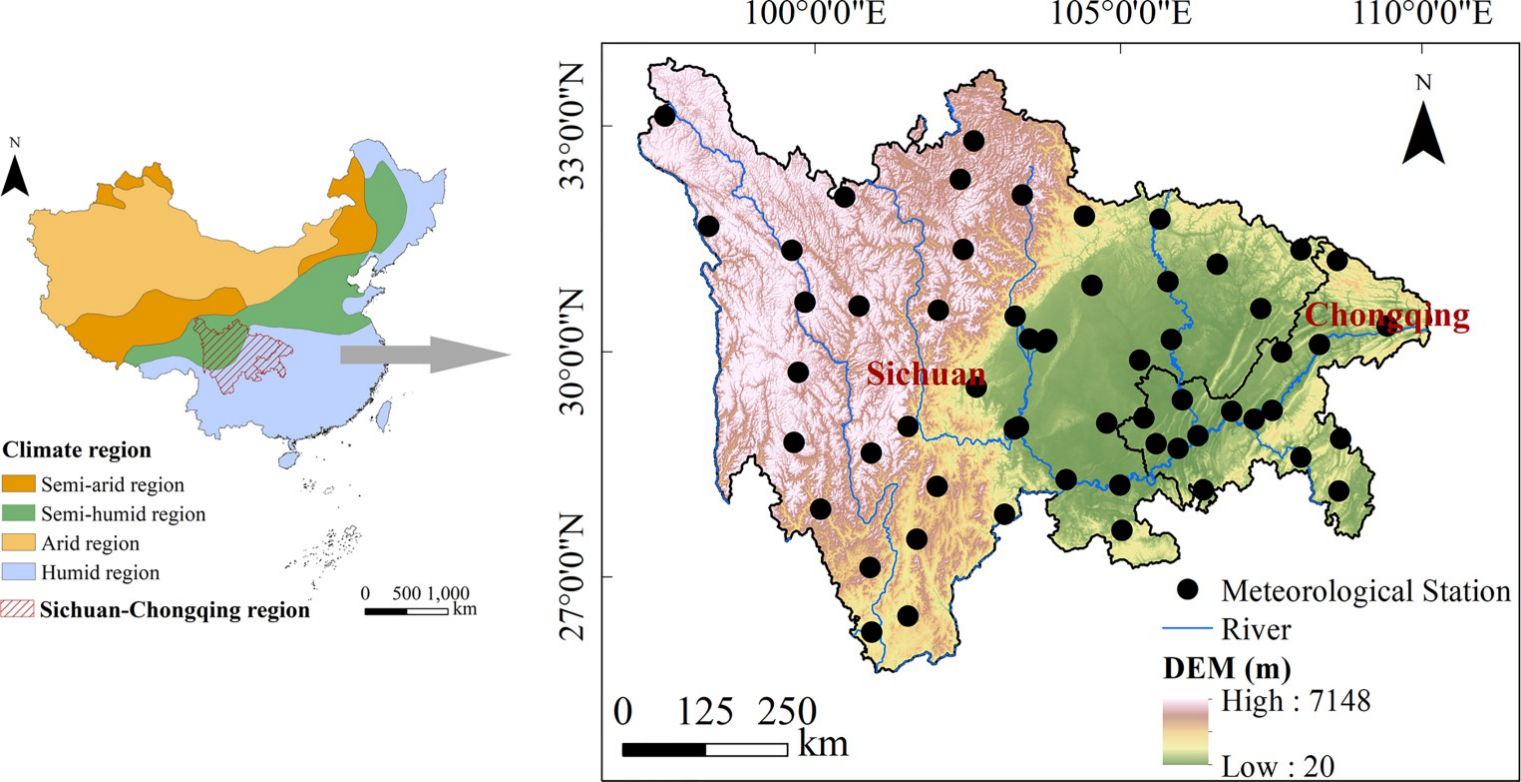
Digital elevation model (DEM) and location of all 56 standard meteorological stations and major rivers in the Sichuan-Chongqing region, China.

The Sichuan-Chongqing region (26°03′–34°19′N, 97°21′–110°11′E; Fig 1) is large (568 402km^2^) and includes 22 cities, including the two megacities Chengdu and Chongqing, which are the principal cities in Southwest China [28]. The region has a complex and diverse climate, controlled by subtropical humid monsoon climate and alpine climate, lying in the semi-humid and humid zone [21, 27]. Annual rainfall varies spatially from 600 to 1700 mm. Multiyear average T_a_ is highly heterogeneous across this region, ranging from −1 to 20°C. The topography of the region is dominated by high mountains, deep basins and river valleys; elevation ranges from 20 to 7148 m, low-lying in the east and high in the west, descending from the northwest to the southeast [27, 29].

Observed daily maximum air temperature (T_max_, °C), minimum air temperature (T_min_, °C), mean air temperature (T_mean_, °C), relative humidity (RH, %), wind speed at 2 m height (U_2_, m/s) and sunlight duration (SD, h) were used to estimate PET. Long term (1970−2020) daily meteorological data from 56 meteorological stations in the SC-CQ region were provided and quality controlled by the China Meteorological Administration (http://www.cma.gov.cn/). The digital elevation model (DEM) of the study area (Fig 1) was provided by the Computer Network Information Center (http://www.gscloud.cn/). Missing data when time gaps were less than five days or greater than five days were respectively filled using linear interpolation and the multiyear mean values of those days [30].

### 2.2 FAO Penman–Monteith model for PET calculation

Various approaches have been developed to quantify PET. The FAO-56 P-M equation [31, 32] was chosen to calculate PET in the current study, because it has gained wide acceptance and is applicable in humid conditions [3, 11–13, 33].

The FAO-56 P-M equation is [10]:

$$\mathrm{PET}=\frac{0.408\Delta (R_{n}‐G)+\gamma \frac{900}{T+273}U_{2}(e_{s}‐e_{a})}{\Delta +\gamma (1+0.34U_{2})}$$ (1)

where *PET* is the daily potential ET rate (mm/d), Δ is the slope of the saturated vapor pressure curve (kPa/°C), *R*_*n*_ is net radiation (MJ/m^2^/d), *G* is soil heat flux density (MJ/m^2^/d; zero on the daily scale), *γ* is the psychrometric constant (kPa/°C), *T* is mean daily air temperature (°C), *U*_*2*_ is mean daily wind speed at 2 m height (m/s), *e*_*s*_ is saturated vapor pressure (kPa), *e*_*a*_ is actual vapor pressure (kPa), and *e*_*s*_−*e*_*a*_ is the vapor pressure deficit (VPD, kPa).

### 2.3 Trend test

The Mann–Kendall (MK) test [34, 35] is highly recommended by the World Meteorological Organization for testing the significance of a hydro-meteorological data trend. The method offers many advantages, including being less sensitive to outlier data [1, 5]. In this study, the nonparametric MK trend test was employed to detect temporal trends in PET and concerned climatic elements for each meteorological station. In order to assess the effects of climate factors on PET, linear regression was used to calculate the trend slope of the climate factors. The slope of the linear regression line represents the mean temporal change in the meteorological factor. If the slope is positive, the meteorological variable shows an upward trend; if the slope is negative, the meteorological variable shows a downward trend.

### 2.4 Contribution and sensitivity analysis of factors controlling PET

Stepwise regression was adopted to quantitatively analyze the contributions of each meteorological variable to variation in PET for each meteorological station in the SC-CQ region during 1970−2020. The meteorological variables were the predictors, and PET was the dependent variable. The stepwise regression can be expressed as:

$$\mathrm{PET}=a_{1}x_{1}+a_{2}x_{2}{+a}_{3}x_{3}+\cdots {+a}_{n}x_{n}+b$$ (2)

where *x*_*1*_, …, *x*_*n*_ are the values of the meteorological variables, *a*_*1*_, …, *a*_*n*_ are the regression coefficients of the meteorological variables, and *b* is the intercept.

The principal factors of change in PET between separate periods can be obtained by the equation:

$$\mathrm{\Delta PET}=a_{1}\Delta x_{1}+a_{2}\Delta x_{2}{+a}_{3}\Delta x_{3}+\cdots {+a}_{n}{\Delta x}_{n}$$ (3)

where *Δx_1_*, …, *Δx_n_* are the trend slopes for each of the meteorological variables, and Δ*PET*, the calculated trend, represents the sum of the contributions of each meteorological variable. The contributions of the meteorological variables (*a_1_Δx_1_*, …, *a_n_Δx_n_*) to change in PET are the product of the meteorological variable trend and the regression coefficient for each meteorological variable [13]. The veracity of this method is tested statistically by comparing Δ*PET* against the PET trends (*Tr*_*PET*_).

In order to evaluate and draw the relative changes of each climatic element against the corresponding relative changes of the PET, a simple but practical method, sensitivity analysis [36], recommended by many previous researchers [7, 11, 16] was chosen in this study.

$$S_{V_{i}}=\underset{\Delta V_{i}\to 0}{\lim}(\frac{\Delta \mathrm{PET}/\mathrm{PET}}{\Delta V_{i}/V_{i}})=\frac{\partial \mathrm{PET}}{\partial V_{i}}∙\frac{V_{i}}{\mathrm{PET}}$$ (4)

where $S_{V_{i}}$ denotes the sensitivity coefficient, and *i* denotes the *i*th variable. A positive (or negative) coefficient represents an upward trend in PET (or decrease) as the climatic variable increase. Assuming all other meteorological variable are constant, if the $S_{V_{i}}$ equals 0.2, 10% increase in meteorological variable would result in a 2% increase in PET. The greater the $S_{V_{i}}$, the higher sensitivity of PET to *V_i_* is expected [16]. In this study, sensitivity coefficients were computed with daily meteorological data. Annual and seasonal average sensitivity coefficients were obtained by averaging daily values.

## 3 Results

### 3.1 Spatiotemporal characteristics of PET

Multiyear mean annual PET from 1970 to 2020 was 872 mm for the entire study area (Table 1). Annual PET showed a significant increasing trend with a rate of 0.78 mm/year (*p* < 0.05). Seasonal variation in PET was also examined in the current study. Multiyear average summer PET had the highest value (369 mm), followed by spring (253 mm), autumn (165 mm) and winter (85 mm). There was an upward trend in PET in all seasons during 1970−2020. Autumn and winter conditions both produced a statistically significant upward trend in PET (*p* < 0.05) as well as an increasing slope with respective gradients of 0.12 and 0.11 mm/year.

**Table 1:** Analysis of seasonal and annual PET throughout the entire SC-CQ region from 1970 to 2020 using the Mann-Kendall test.

	Spring	Summer	Autumn	Winter	Annual
Average value (mm)	253	369	165	85	872
Trend slope (mm/year)	0.17	0.07	0.12 *	0.11 *	0.78 *

Note: * indicates the 0.05 significance level.

**Fig 2:**
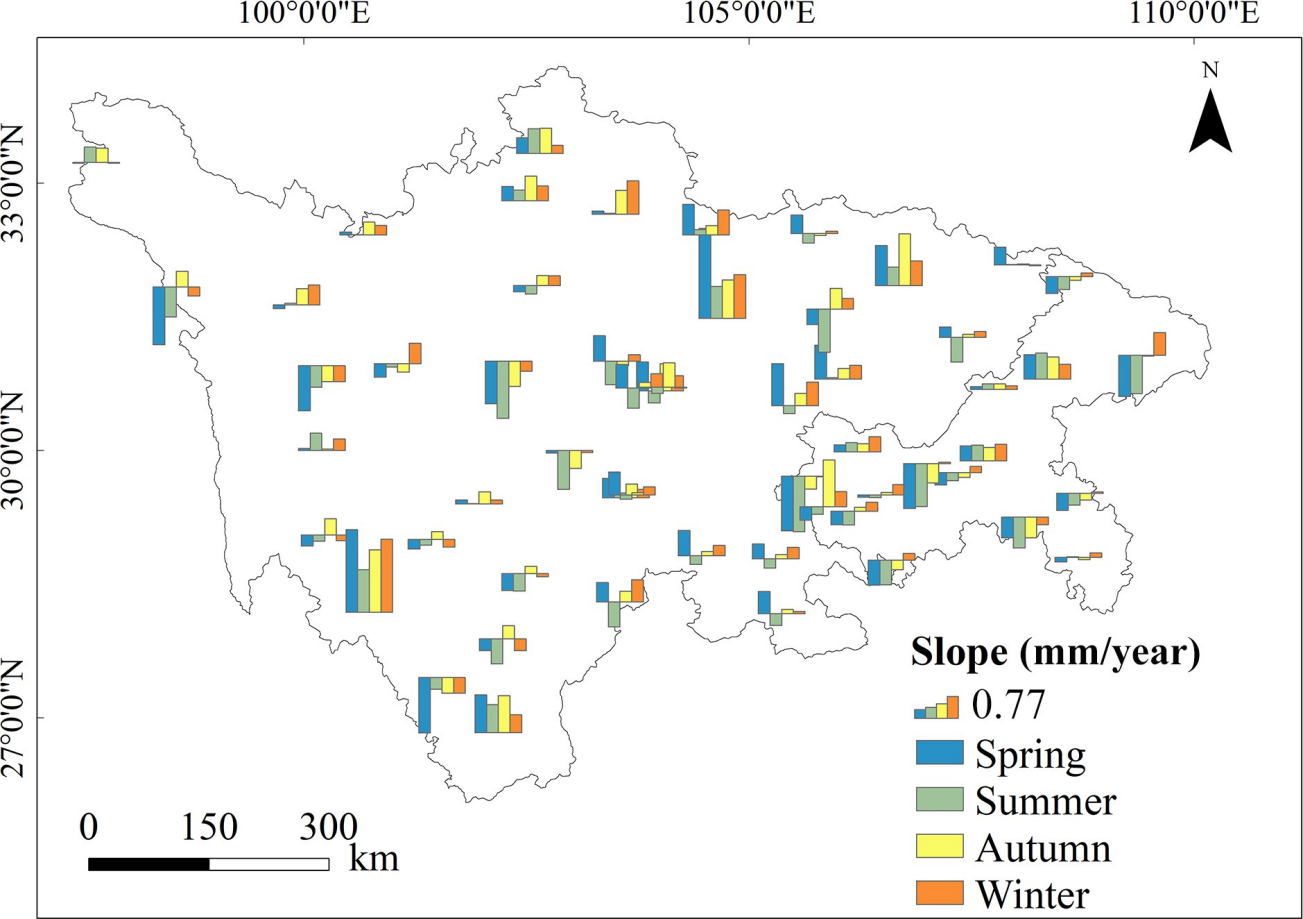
Seasonal trends of potential evapotranspiration at each station during 1970−2020. The columns are scaled according to the magnitude of the trend. The upward and downward columns denote increasing and decreasing tendencies, respectively.

Seasonal PET trends were calculated for each station to show spatial variation in PET (Fig 2 and Table 2). PET did not vary uniformly across the study area in different seasons. Although summer PET exhibited an increasing trend from 1970 to 2020, 35 stations showed negative trends in summer PET, 11 of which were significant decreases. This phenomenon implied that consideration only of average regional PET is inadequate to reveal the mechanisms that triggered PET variation. Of the 56 sites analyzed, 18 sites exhibited significant positive PET trends (*p* < 0.05) in autumn and 12 in spring, whereas significant negative trends (*p* < 0.05) were observed in only three sites in autumn and seven in spring. The greatest number of sites with increasing PET trends was found in winter (about 75% of all sites); 15 sites were statistically significant (*p* < 0.05).

**Table 2:** Numbers of stations with different trends of potential evapotranspiration by season.

	Spring	Summer	Autumn	Winter	Annual
Significant positive trend (*p* < 0.05)	12	4	18	15	12
Nonsignificant positive trend	26	17	23	26	21
Significant negative trend (*p* < 0.05)	7	11	3	4	8
Nonsignificant negative trend	11	24	12	11	15

**Fig 3:**
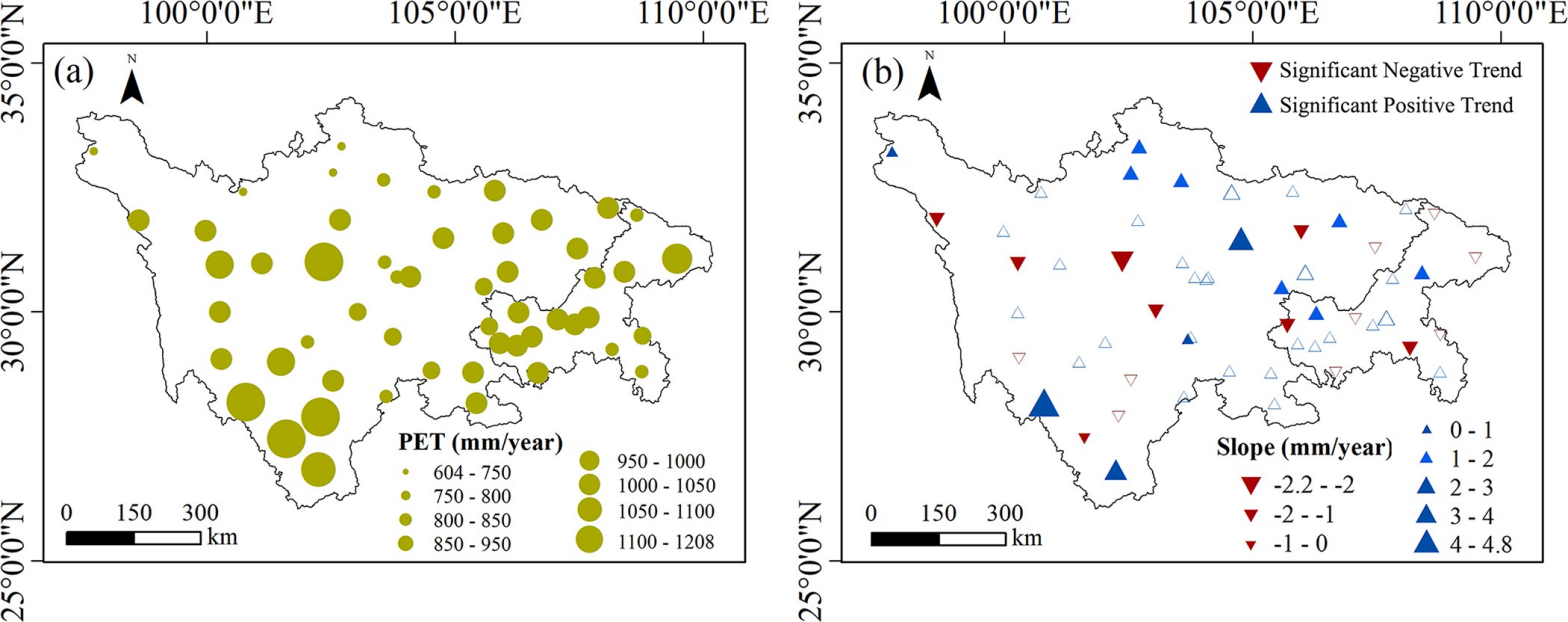
Spatial distributions of (a) mean annual PET, and (b) its trends slopes and the MK test results (*p* < 0.05) in the Sichuan-Chongqing region for 1970–2020. Blue upward and red downward triangles represent positive and negative trends, respectively. Solid triangles indicate trends are statistically significant (*p* < 0.05).

Multiyear average PET across the entire SC-CQ region in 1970–2020 ranged from 600 to 1200 mm. The annual distribution showed a coherent spatial pattern, with a relatively low value in the north of the study area and a relatively high value toward the south (Fig 3(A)). The trends for annual PET at 56 stations in the period 1970−2020 are shown in Fig 3(B). Annual PET showed a positive trend at 33 stations (58.9%), mainly in the northern part of the area; the trend was significant at 12 stations at a 95% confidence level. A negative trend in PET was found at 23 stations (41.1%); the trend was statistically significant at eight stations (14.3%) at a 95% confidence level. Change in PET exhibited an uneven distribution, which indicated that PET variation was arose from the combined effect of meteorological elements.

### 3.2 Spatiotemporal characteristics of basic meteorological factors

**Fig 4:**
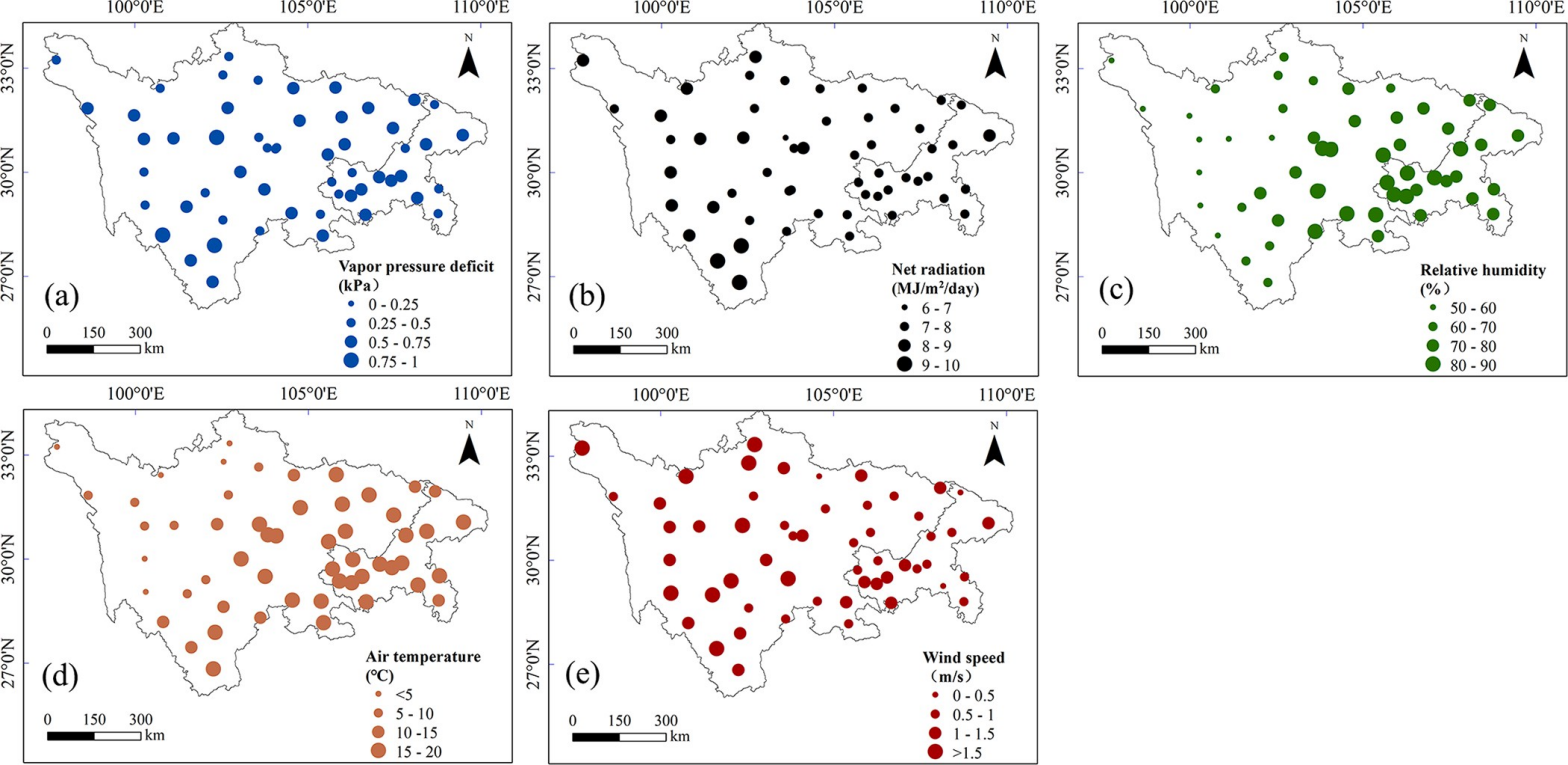
Mean annual values of five basic meteorological variables ((a) vapor pressure deficit, (b) net radiation, (c) relative humidity, (d) air temperature and (e) wind speed) at each station in the Sichuan-Chongqing region from 1970 to 2020. Republished under a CC BY license, with permission from the China Meteorological Data Service Center, original copyright 2005–2017.

**Fig 5:**
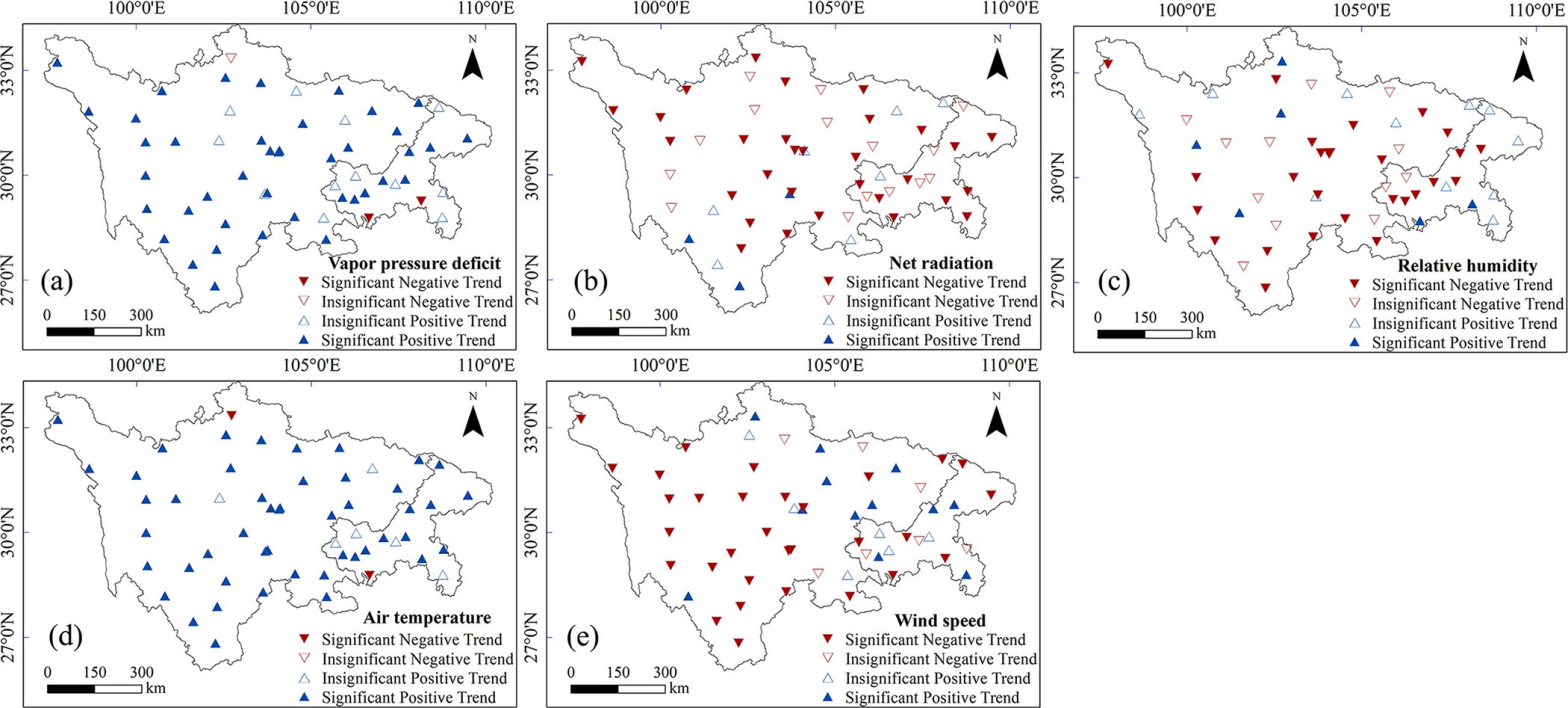
Spatial distributions of changes in climatic factors ((a) vapor pressure deficit, (b) net radiation, (c) relative humidity, (d) air temperature and (e) wind speed). Blue upward and red downward triangles represent positive and negative trends, respectively. Solid triangles indicate trends are statistically significant (*p* < 0.05).

**Fig 6:**
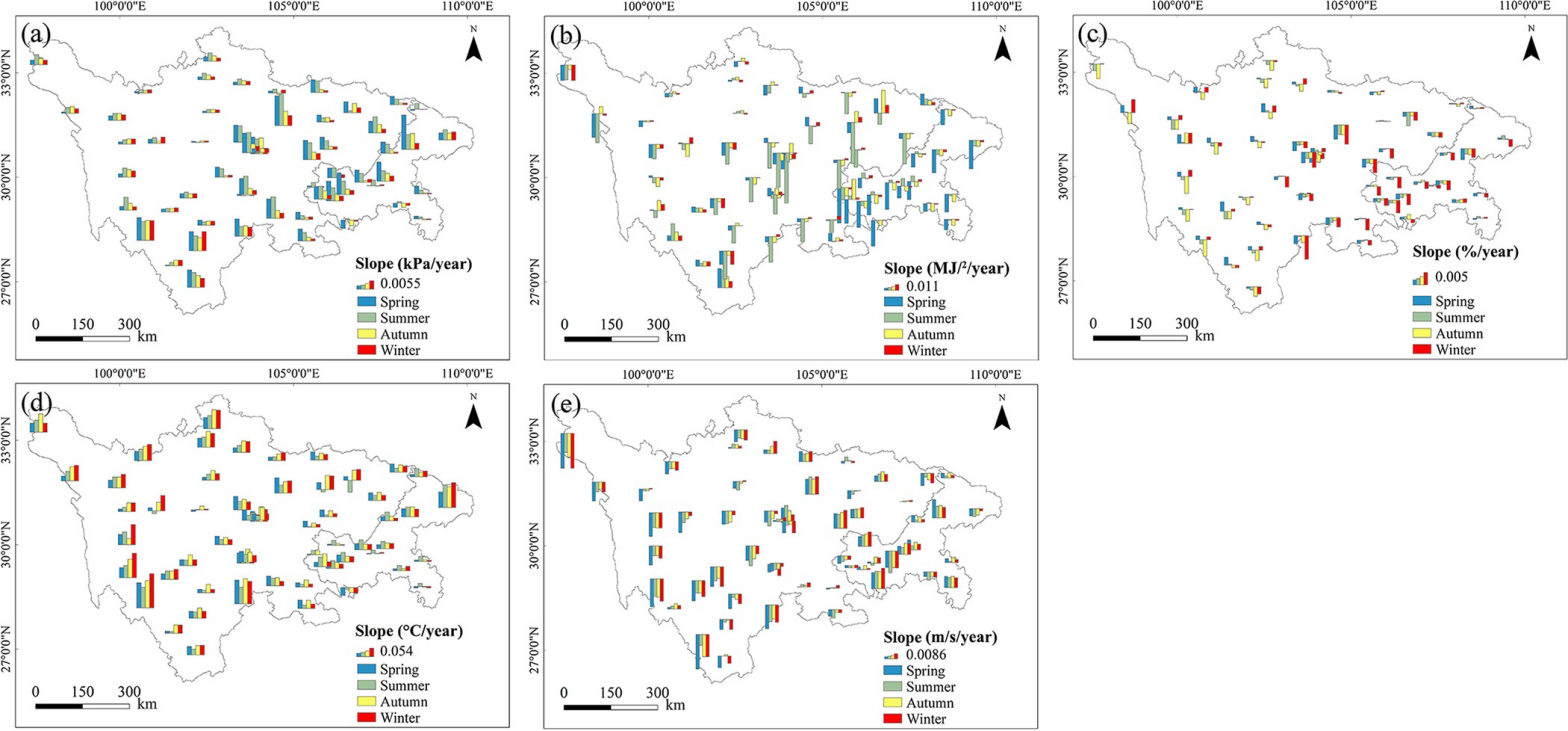
Same as in Fig 2, but for the five meteorological variable: (a) vapor pressure deficit; (b) net radiation; (c) relative humidity; (d) air temperature; (e) wind speed.

**Fig 7:**
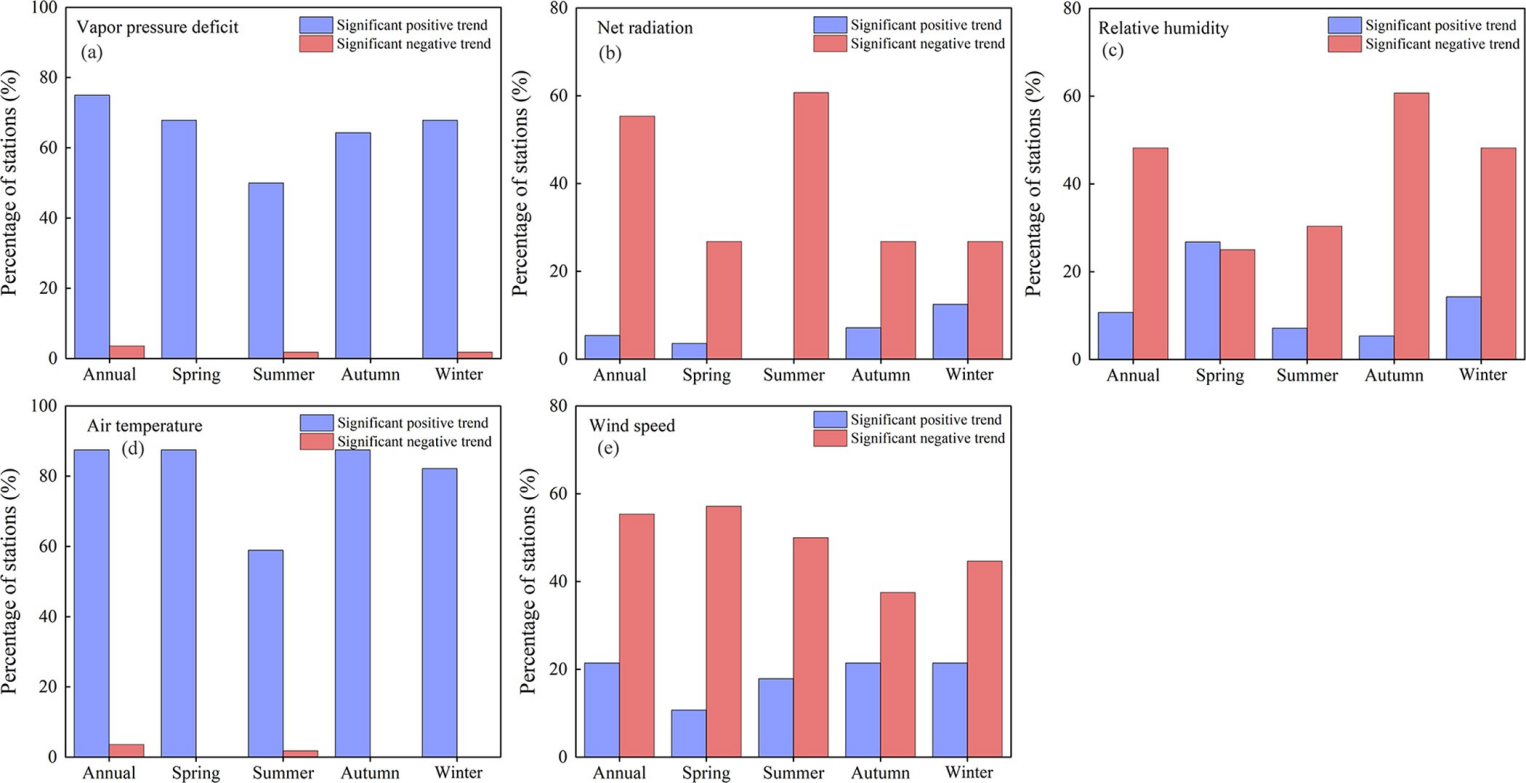
Percentage of stations with significant trends (*p* < 0.05) for five basic meteorological variables ((a) vapor pressure deficit, (b) net radiation, (c) relative humidity, (d) air temperature and (e) wind speed) at annual and seasonal scales.

The same trend analysis was conducted at both annual and seasonal scales to identify the trigger mechanisms of PET variation. Wind speed (W_s_), air temperature (T_a_), relative humidity (RH), net radiation (R_n_) and vapor pressure deficit (VPD) were analyzed in this study (Figs 4–7). Mean annual T_a_ and mean annual RH had fairly similar spatial distributions, with a clear difference between east and west and decreasing from the southeast to the northwest (Fig 4). Mean annual R_n_ showed a clear southwestern–northeastern high-low gradient. Compared with other meteorological variables, mean annual VPD and mean annual W_s_ showed complex spatial patterns. Relatively low mean annual VPD and mean annual W_s_ values were primarily scattered in northwestern and northeastern areas of the SC-CQ region.

Annually, spatial trends for five meteorological variables were also identified in this study (Fig 5). Positive trends of mean annual VPD were found across the entire study area except for some negative trends at a few isolated sites. A similar pattern was found in the annual T_a_. Over 85% of the sites showed a positive trend for annual T_a_ (*p* < 0.05), most of which (>50% of all stations) were statistically significant. This result suggests the general sharp warming in the study area, which is in line with the global warming trend detected in many areas of the world. Annual R_n_ and annual RH were characterized by negative trends across the study area except at a few sites. Changes of annual W_s_ had strong spatial variabilities. An upward trend was found mainly in the east of the study area and a downward trend was found in the western and central areas.

Seasonally, the spatial distributions of trends of the five meteorological factors are shown in Fig 6. The percentages of stations showing statistically significant trends (*p* < 0.05) for these variables are displayed in Fig 7. The overall impression was that the changing direction of seasonal meteorological factors and their distributions were generally consistent with those at the annual scale, shown in Fig 5. Seasonal and annual average VPD both showed a significant positive trend at most stations. Significant negative trends of VPD were not detected in spring and autumn. Rather weak change trends were found in the northwest of the study area.

The number of stations with statistically significant negative trends in R_n_ were considerably more numerous than that with statistically significant positive trends. Annually average R_n_ showed a significant negative trend for 31 stations (55% of all stations). In seasonal distributions, the maximum number of significantly decreasing trends in average R_n_ was found in the summer. Compared with other meteorological factors, there was no clear seasonal variation in RH between different stations, even though some stations showed statistically significant change trends.

The warming trends for both seasonal and annual T_a_ were overwhelming, and most sites showed a significant increasing trend (*p* < 0.05). Winter T_a_ had the largest trend slopes when compared to other seasons. In contrast with T_a_, both annual and seasonal W_s_ showed a significant decreasing trend at most stations. Stations that showed a negative trend in W_s_ were mainly in the western part of the study area.

In general, on both an annual and a seasonal basis, trends in these basic meteorological factors were spatially very variable. However, a dominance of downward trends in R_n_ and W_s_, and upward trends in T_a_ and VPD could be seen in this region. Average RH showed a spatial mix of increasing and decreasing trends both annually and seasonally, with more apparent downward trends.

### 3.3 Dominant meteorological factors

#### 3.3.1 Quantitative PET contribution analysis

**Fig 8:**
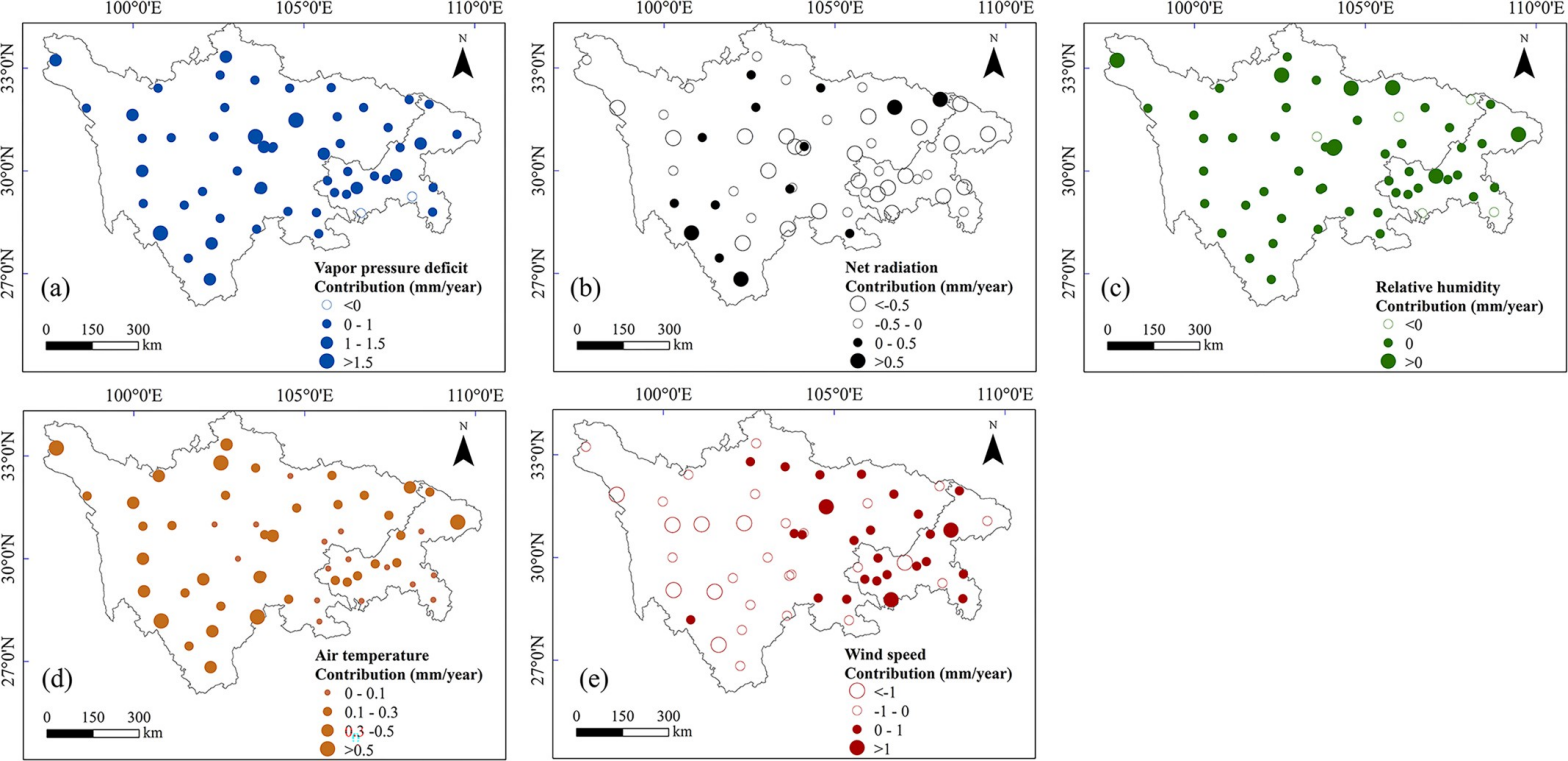
Spatial distributions of contributions of five basic meteorological factors ((a) vapor pressure deficit, (b) net radiation, (c) relative humidity, (d) air temperature, and (e) wind speed) to PET trends at an annual scale.

The contributions of different driving factors using stepwise regression and compared the impacts of different factors for each meteorological station were investigated in the study area. The coefficient of determination (*R*^2^) between Δ*PET* and *Tr*_*PET*_ was 0.9. This high correlation implied that stepwise regression was able to identify the effects of the meteorological factors on PET trends. Fig 8 displayed the contributions of five meteorological elements (VPD, R_n_, RH, T_a_ and W_s_) to the mean annual PET trends and the spatial distribution of their importance.

(1) VPD had motivated efforts to annual PET change for almost all stations, with contribution value ranging from −0.4 to 2.5 mm/year. The center of Chongqing province and center and northwest of Sichuan province achieved relatively greater contribution value. (2) R_n_ was strongly related to PET at an annual scale (average contributions varied from −1.8 to 1.2 mm/year). The contributions of R_n_ on PET on an annual scale were negative for most stations. (3) The contributions of RH on PET were close to zero for more than half of the stations, which indicates that changing effects of RH on annual PET were weakened for this study area when compared to other climatic factors. (4) The station to station variability of contribution of T_a_ on PET trend was in the range of 0 to 1.1 mm/year. One salient feature was that the contributions of T_a_ to PET on the periphery of the study area were relatively high. (5) The positive annual contributions of W_s_ to PET were mainly distributed in the eastern part of the study area and negative contributions were mainly found in the western region. Contributions of annual W_s_ to PET were in the range −2 to 1.4 mm/year. Over the entire region, the contributions of decreased W_s_ and R_n_ were balanced by increased T_a_ and VPD, leading to increased PET.

**Fig 9:**
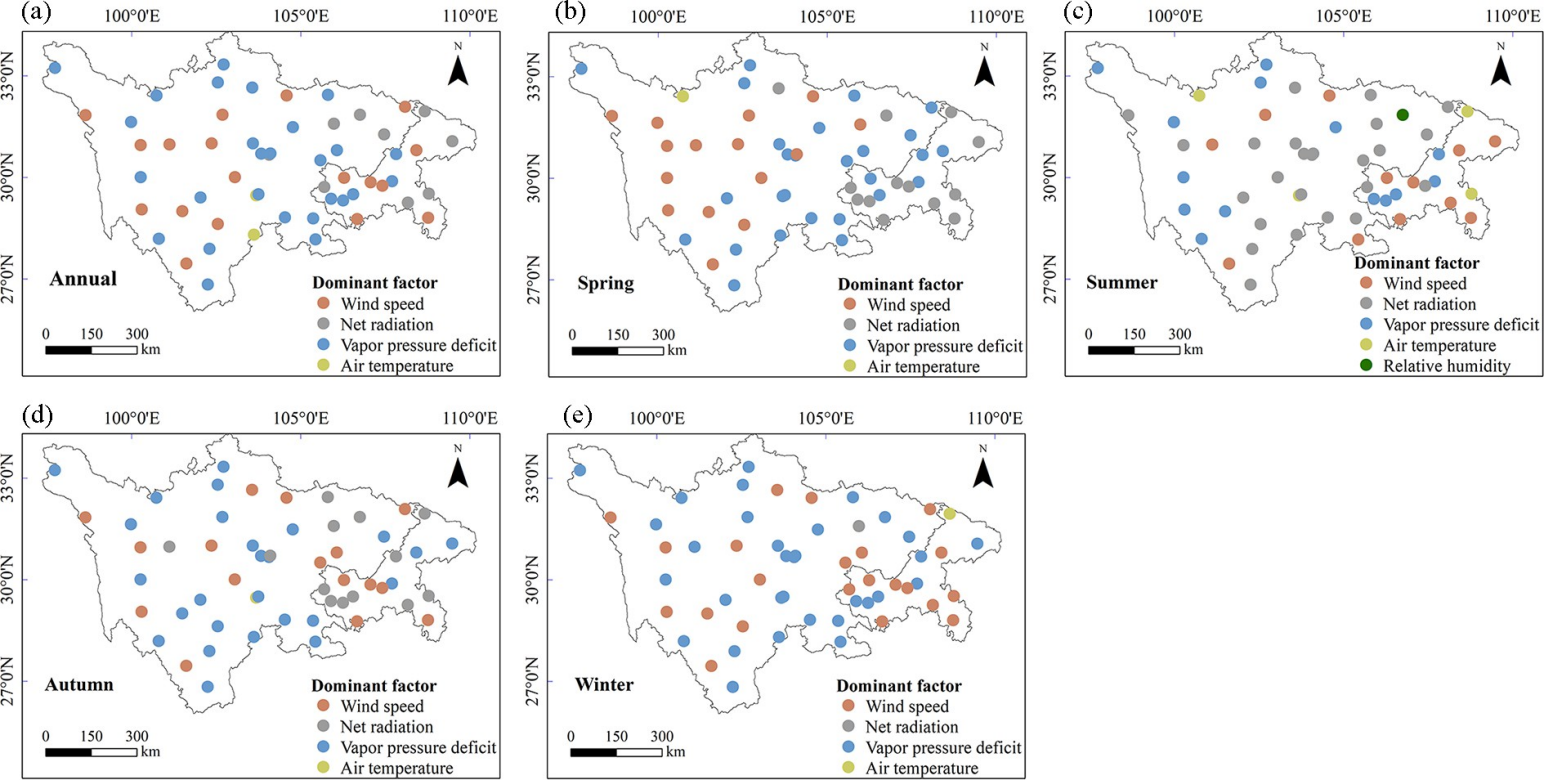
Spatial distributions of dominant factors of the change in PET in (a) annual, (b) spring, (c) summer, (d) autumn and (e) winter during 1970−2020.

**Fig 10:**
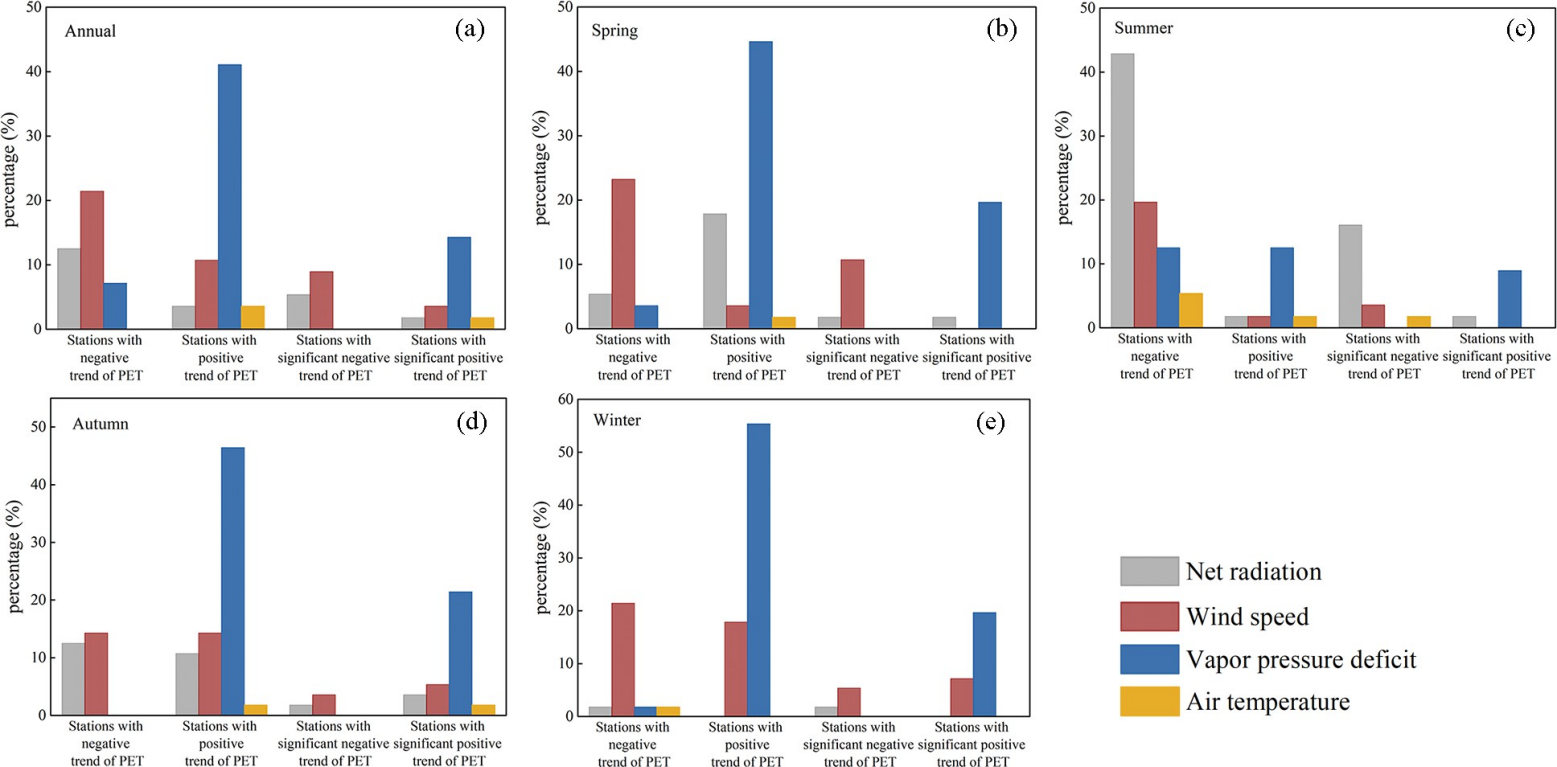
Dominant factors are shown as percentages for stations with negative, positive, significantly negative (*p* < 0.05) and significantly positive (*p* < 0.05) trends of PET in (a) annual, (b) spring, (c) summer, (d) autumn and (e) winter.

Fig 9 presents the spatial distribution of the leading meteorological factors of PET, and Table 3 lists the numbers of stations at which a particular climatic factor was dominant at annual and seasonal scales. In terms of annual effects, the distribution of climate factors varied spatially (Fig 9). Given that VPD caused changes in PET at 27 stations (close to 50%), it was regarded to be most strongly associated with the variation in annual PET (Fig 10). Changes in annual PET in the center and west of the study area were mainly attributed to VPD and W_s_. R_n_ dominated changes in annual PET was observed in the east of the study area. VPD was the major driver of increasing PET trends, followed by W_s_. W_s_ was the major driver of decreasing PET trends, followed by R_n_. In contrast to the other four meteorological factors, RH had little effect on annual PET across the SC-CQ region.

**Table 3:** Number of stations with dominant factors of PET trends at annual and seasonal scales during 1970−2020.

Time scale	Dominant factors				
	VPD	R_n_	RH	T_a_	W_s_
Annual	27	9	0	2	18
Spring	27	13	0	1	15
Summer	14	25	1	4	12
Autumn	26	13	0	1	16
Winter	32	1	0	1	22

In seasonal terms, the dominant factor contributing to changes in PET varied across stations. There were clear regional differences in spring. W_s_ was the dominant factor in the northwest. VPD was the dominant factor in the southeast. In summer, R_n_ was the factor most strongly associated with decreased PET, and VPD was mainly responsible for increased PET. Summer PET was greatly affected by changes in R_n_, followed by VPD and W_s_. In autumn, stations at which VPD was the dominant driver of PET were located mainly in the central–western area, and those at which R_n_ was the dominant driver were located mainly in the eastern area. In winter, VPD had the strongest positive effect on PET, and W_s_ mainly controlled the decrease in PET. Stations at which VPD or W_s_ were dominant factors were widely dispersed across the study area. VPD had an overwhelmingly dominant effect on the increase in PET at most stations in most seasons, and RH contributed least. Except in summer, W_s_ was generally the dominant driver for inducing the decreasing PET trend.

#### 3.3.2 Quantitative PET sensitivity analysis

**Fig 11:**
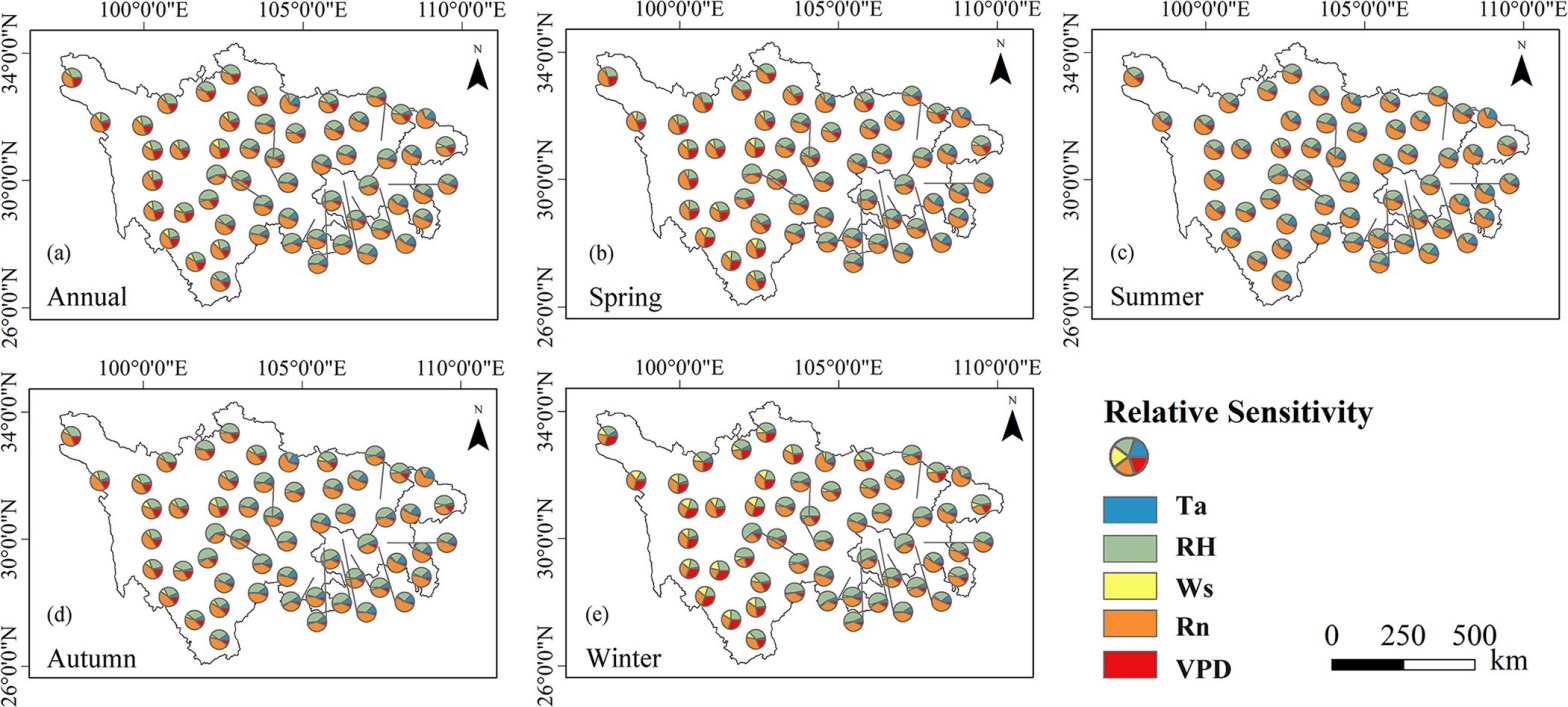
The relative sensitivity of the PET to air temperature (T_a_), vapor pressure deficit (VPD), net radiation (R_n_), relative humidity (RH) and wind speed (W_s_) in annual (a), spring (b), summer (c), autumn (d) and winter (e) during 1970–2020 at 56 stations.

To better understand the influence of climatic variables on the PET trend, the sensitivity analysis of the PET to climatic variables was performed. The averaged sensitive coefficients of PET to five basic meteorological variables of all observation sites are shown in Table 4 and the spatial patterns of the relative sensitivity of the PET to five basic meteorological elements are displayed in Fig 11. The combination of Table 4 and Fig 11 explains the result of sensitivity analysis. Annual and seasonal PET were positively correlated with T_a_, R_n_, VPD, and W_s_, and negatively correlated with RH (Table 4), indicating an increase (or decrease) in PET as the meteorological factors increase (or decrease) except for RH. On a full-year basis, although the spatial pattern of the relative sensitivity illustrated that the PET response to changes in each meteorological factor was different from site to site. R_n_ and RH were the two most sensitive elements on annual PET throughout the whole study region in general, followed by VPD. The least sensitive variable was W_s_. The highest absolute sensitivity coefficient was computed for T_a_ (0.19), VPD (0.29), R_n_ (0.85), RH (0.70) and W_s_ (0.15) in summer, winter, summer, winter and winter, respectively. The most sensitive variable on spring (0.75), summer (0.85) and autumn (0.80) PET was R_n_. During winter, the greatest sensitivity of PET was for RH (−0.70).

**Table 4:** The sensitivity coefficient of the potential evapotranspiration to the five basic meteorological variables.

Time scale	Sensitivity coefficient				
	*S*_*Ta*_	*S*_*VPD*_	*S*_*Rn*_	*S*_*RH*_	*S*_*Ws*_
Annual	0.14	0.21	0.78	−0.6	0.10
Spring	0.13	0.23	0.75	−0.53	0.09
Summer	0.19	0.14	0.85	−0.49	0.05
Autumn	0.16	0.19	0.80	−0.69	0.09
Winter	0.06	0.29	0.63	−0.70	0.15

Note: S_Ta_, S_VPD_, S_Rn_, S_RH_ and S_Ws_ denote the sensitivity coefficient of potential evapotranspiration to air temperature (T_a_), vapor pressure deficit (VPD), net radiation (R_n_), relative humidity (RH) and wind speed (W_s_), respectively.

## 4 Discussion

### 4.1 PET trend

Various studies have quantified PET trends in many regions over different periods [13, 15, 18, 37]. Most studies, however, have focused on regional average PET trends and taken no account of the spatial distribution of PET. In a large region with unique weather conditions and complex topography like the SC-CQ region, long-term trends in annual and seasonal PET and its primary driving mechanisms were inevitably spatially variable, which have not been fully analyzed in published research [15]. We examined the regional diversity of the PET in the SC-CQ region during 1970−2020. It can be found from the above analysis that there were complex spatial patterns in the PET and its change. Overall, at almost 60% of the meteorological stations in the region, mainly distributed in the north, annual average PET showed a positive increasing trend (Fig 3(B)). Similar to our study, high spatiotemporal heterogeneity in PET has also been found in the Yellow River basin [15] and the Beijing-Tianjin Sand Source Control Project region [12], both of which are large regions in China.

Some studies have found that PET showed a distinct negative trend along with significantly increasing T_a_, and identified this phenomenon as an *evaporation paradox*. In the SC-CQ region, on a seasonal scale, more sites showed an increasing trend than a decreasing trend, except in summer, which confirmed the existence of the *evaporation paradox* generally in the months of summer. On a regional scale, the *evaporation paradox* phenomenon occurred more frequently in the southern Sichuan-Chongqing region during 1970–2020. Similar findings reported an *evaporation paradox* accompanied by the simultaneous PET decrease and T_a_ rise within China (e.g. Yangtze River catchment [38] and Beijing-Tianjin region [12] and some other regions worldwide [39, 40]. Another form of the *evaporation paradox* expressed by a declined T_a_ and an elevated PET was also reported by previous studies [41]. A possible explanation of these two forms of the *evaporation paradox* is that the increased T_a_ and the decreased W_s_ or sunshine duration have the first role in the PET reduction and the increased atmospheric demand mainly cause the increase of PET. These spatial and regional differences of the PET response to meteorological variables need further investigation.

### 4.2 Meteorological factors of PET variation

#### 4.2.1 Contribution and sensitivity of PET to the change of meteorological factors

Long-term trends of annual and seasonal PET are directly influenced by the combined effects of changes in meteorological variables such as wind speed, radiation and humidity [1, 7]. The individual contribution of climate factors to PET was identified in the current study. Decreases in W_s_ and R_n_ and increase in VPD greatly affected the changing direction of PET. The results we obtained that identified the factors having the most effect on PET are generally consistent with those from previous studies of other regions, such as Siberian river basins [11] and the Qinhuai River basin [3, 4]. Spatial distributions of driving factors were complex in the study region (Fig 9). Differences across the region were primarily due to the study area being large and having a complex topography and a heterogeneous climate. Similar characteristics have been studied in many parts of China, such as the Yellow River basin [15], the Yangtze River basin [38], and the Haihe River basin [1].

In order to further quantitatively assess the variability of PET to the changes in the controlling meteorological factors across the entire study area, conducting sensitivity of PET to meteorological elements at annual and seasonal scales is necessary. The positive sensitivity coefficient for T_a_, VPD, R_n_ and W_s_ indicated the PET increases with the increase of these meteorological elements, while the negative sensitivity coefficient for RH implied the PET increases with the decrease of RH (Table 4). PET was highly sensitive to R_n_ and RH relative to the other basic meteorological variables in this study area. The results of sensitivity analysis in this study are close to the previous fundings. Numerous studies concluded that RH was the most sensitive factor on PET in southwest China [42], Yellow River Basin [15] and coastal southern Iran [16]. Xu et al. (2006) indicated that PET was most sensitive to the R_n_, followed by RH, air temperature and W_s_ in Yangtze catchment. R_n_ and RH were considered as the most-second sensitive factor in Qilian Mountains, northwestern China [43]. The differences of the most sensitive variable existed in some regions, e.g., Guo et al. (2017) found that air temperature had the highest sensitivity in Austrailia.

Averaged across all meteorological stations, the greatest and lowest sensitivity coefficient of PET to T_a_ (S_Ta_) was found in summer (0.19) and winter (0.06), respectively. It is in agreement with our common sense that the warmer the T_a_ is, the more the sensitivity of PET to T_a_ is [15]. Additionally, our results addressed that W_s_ appeared to cause minor perturbation in PET change in the study area. However, the current studies conducted in the arid region revealed that the sensitivity of PET to W_s_ was considerable [16, 40]. The difference may be attributed to the different climates in the study area.

The impacts of meteorological factors were dependent not only on the sensitivity of PET to meteorologcial factors, but also on the change magnitude of meteorological factors [15, 38, 42]. Although RH exhibited high sensitivity to PET at annual and seasonal scales, it had little effect on variation in PET during the study period because of its much smaller change magnitude relative to other climatic elements. Similar to fundings by Xu et al. (2006), they proposed that RH produced little contribution to changing PET, despite it was the most sensitive variable in the Yangtze River catchment. Moreover, a relatively low S_Ws_ was found in the SC-CQ region, while changes in W_s_ were found to play a significant role in the PET changing as the W_s_ of most stations showed a significant trend (Fig 5). The VPD was one of the driving factors affecting the PET variation because it was not only the sensitive variable, but also a variable with a significant positive trend at most stations across the whole study region. Presenting sensitivity and contribution of meteorological elements to PET simultaneously not only can avoid uncertainties from a single approach, but also provide comprehensive interpretation for PET dynamic over this region [15].

#### 4.2.2 Impacts of meteorological factors on trends of PET

The globally warming climate has accelerated the water cycle, which has further increased terrestrial PET [5, 17, 44]. In our study, T_a_ showed increasing trends in the entire study period and winter T_a_ had the largest trend slopes for most stations, which is consistent with the persistent warming in winter currently observed in China [15]. T_a_ has increased globally in response to the increase in atmospheric greenhouse gases [14], increased haze formation [29, 45], and increased cloud cover [3]. Some studies have identified significant heat island effects in SC-CQ due to urban development [46]. The complex topography makes the local climate more responsive to land surface changes and increased pollution emissions [29], and so the increase in T_a_ in this region was inevitable. However, an increase in T_a_ was not the primary driver of the increase in PET in all cases. T_a_ had much less effect on annual and seasonal PET than W_s_, VPD or R_n_ for most stations in SC-CQ. Similar results have been obtained in many previous studies of other regions worldwide [3, 12, 37, 40]. However, Darshana et al. (2013) used sensitivity analysis and found that change in annual PET across the Tons River basin in central India was caused mainly by changes in maximum T_a_. The inconsistency between their findings and ours may be due to differences in the study period and study regions.

In a humid atmosphere, solar radiation is the principal source of energy required for evaporation [47]. Thus the decrease in R_n_ has been considered to be a key driver of the decrease in PET and has been found to be the case for many regions across the world, such as the Taohe River basin [48], the Hai River basin [37], the entire northern hemisphere [49], and northeast India [39]. A gradual decrease in solar radiation or sunlight duration is likely caused by increased air pollution [47] and change in W_s_ [50]. Increased levels of aerosols from contaminants have resulted in increased cloud cover, which had a negative effect on sunshine duration and available solar radiation [51]. W_s_ is highly dependent on solar radiation because wind is caused by radiation-induced differences in surface temperature [50]. Thus, interactions between W_s_ and aerosols have a strong effect on radiation, and then drive changes in PET.

For the whole study area, W_s_ was a substantial factor affecting the PET trend, particularly in spring and winter. Other research has also attributed the decreased PET to W_s_, for example in the Jing River basin [7], the Qinhuai River basin [3], an arid region of China [14], and northeast India [39], among other regions. Annual and seasonal W_s_ showed decreasing trends at most sites during 1970–2020 (Figs 5 and 6), which suggests a weakening of the East Asian monsoon in the SC-CQ region. The reduction of W_s_ can be greatly induced by various causes including regional and global warming [52, 53], human-induced factors [4, 5, 54], and changes in atmospheric circulation patterns [7]. Under the background of vigorously developing western China, SC-CQ has thus experienced extensive rapid urbanization and population growth in recent decades [25]. Some studies have suggested that a decrease in W_s_ was at least partially correlated to an increase in surface roughness caused by human activity [13, 55]. It has been suggested that the reduced surface pressure gradient between high and low latitudes in southwest China might be a reason for decreased W_s_ [56]. The issue of what drives change in W_s_ is clearly complex, and so the cause of the decrease in W_s_ across SC-CQ needs more investigation.

Increased VPD was the major driver of change in PET at all time scales. Increase in VPD countered the effects of decreases in W_s_ and R_n_ on PET, leading to a positive PET trend across the entire study area. VPD appears to have been the principal factor in increasing the rate of moisture cycling and evapotranspiration. Atmospheric water demand, which is closely related to VPD, was also responsible for the change in PET in this region [3, 44, 57]. Our results are consistent with those obtained by Qin et al. (2019), Tang et al. (2021), and Abtew et al. (2011), which respectively show that VPD was a dominant climate factor of increased PET in southern China, Siberian river basins, and southern Florida. Hao et al. (2018) found that VPD was positively correlated with T_a_ and negatively correlated with RH in a wet region. These results suggest that the increasing PET trend may be more rapid for humid areas, where increasing T_a_ and decreasing atmospheric humidity induce increase in VPD. Researchers worldwide have recently become more interested in the effects of change in VPD [19, 40, 44]. Vast quantities of research highlighted the elevating VPD and the consequent increased PET have identified significant effects on the water cycle [40] and the carbon cycle [57], which can potentially exacerbate water stress and existing urban heat island effects [44, 58].

### 4.3 Deficiencies of this study

This study examined the spatio-temporal pattern of the PET as well as of the climatic elements that affect PET in the Sichuan-Chongqing region from 1970 to 2020. However, its main drawbacks include:

1. Long-term weather data gathered from meteorological observation sites for calculating PET have been widely used in similar studies [5, 12, 16, 38]. The strength of the observational data at certain sites is that it may reflect the influence of local climate effects. However, it may not represent the spatial variability of weather over a large study area due to the density of weather stations. Gridded weather data provide more comprehensive climate information, and they have been used for estimating PET within the whole region recently [59]. It should be noted that some researchers maintained that gridded weather data sets could overestimate PET results, especially in well-watered surface, owing to the chronic overstatement of T_a_ and W_s_ and understatement of air humidity [59–61]. The selection of different data sets for the regional-scale PET evaluation would have an impact on the results. Future studies can be carried out to estimate trends of PET and its driving factors using the gridded datasets, and evaluate the performance of gridded and weather station data.
2. In addition to the five basic meteorological factors analyzed in this paper, some other variables (e.g. sky cover and sunlight duration) can indirectly affect the spatio-temporal pattern of the PET trend [1, 13, 54]. Future studies should be further explored these parameters as major controlling factors to fully account for PET change.

## 5 Conclusions

The FAO-56 Penman–Monteith model was adopted to analyze long-term (1970−2020) PET time series data. The contributions of variation in net radiation (R_n_), wind speed (W_s_), air temperature (T_a_), vapor pressure deficit (VPD) and relative humidity (RH) to the PET trend were quantitatively assessed in 56 stations across the Sichuan-Chongqing region over the past 51 years. The PET sensitivity to these meteorological variables was also estimated in this study. Key findings from this study are summarized as follows.

1. Seasonal and regional differences in PET and their associated meteorological variables are bound to exist. There was a positive trend in PET for more than 58% stations at annual scale. Approximately 68%, 38%, 73% and 73% of all surveyed weather stations produced an increasing trend in PET during spring, summer, autumn and winter, respectively. The annual W_s_ trend across the study area varied spatially, with a negative trend in the east of the study area and a positive trend in the west and center. Most stations showed increasing trends in T_a_ and VPD, and decreasing trends in W_s_ and R_n_ on an annual scale. Annual RH showed a mixture of increasing and decreasing trends spatially, with the decreasing trends being stronger. Spatial heterogeneity was also observed in the seasonal trends of meteorological variables.
2. Climate change has greatly impacted local hydrology. Contribution analysis showed that W_s_ was the primary controlling factor in PET in annual, spring, autumn and winter, and R_n_ was the primary influence in summer for stations with a negative PET trend. VPD was the dominant factor in all time scales for stations with a positive PET trend. The positive effects of VPD offset the negative effects of W_s_ and R_n_, leading to increased PET for the entire study area. Sensitivity analysis revealed that annual and seasonal PET were positively correlated with all climatic variables except for RH. PET was mostly sensitive to RH and R_n_. The results from the contribution approach did not coincide with those of the sensitivity analysis. A comparative analysis illustrated that the effects of meteorological factors are influenced by not only the sensitivity of PET to meteorological factors but also the change magnitude of the meteorological factors.
3. The distinct seasonal and regional characteristics of changes in PET and their causes showed the importance of estimating the hydro-meteorological processes at fine spatiotemporal resolution. VPD and R_n_ as the main causes of PET changing must be taken into account for the water cycle and local natural resources in an area of climate change and intense human activity.

## Supporting information

S1 File (XLSX)
